# Etodolac Fortified Sodium Deoxycholate Stabilized Zein Nanoplatforms for Augmented Repositioning Profile in Human Hepatocellular Carcinoma: Assessment of Bioaccessibility, Anti-Proliferation, Pro-Apoptosis and Oxidant Potentials in HepG2 Cells

**DOI:** 10.3390/ph15080916

**Published:** 2022-07-24

**Authors:** Ahmed K. Kammoun, Maha A. Hegazy, Alaa Khedr, Zuhier Ahmed Awan, Maan T. Khayat, Majid Mohammad Al-Sawahli

**Affiliations:** 1 Department of Pharmaceutical Chemistry, Faculty of Pharmacy, King Abdulaziz University, P.O. Box 80260, Jeddah 21589, Saudi Arabia; akammoun@kau.edu.sa (A.K.K.); akhedr@kau.edu.sa (A.K.); mkhayat@kau.edu.sa (M.T.K.); 2 Analytical Chemistry Department, Faculty of Pharmacy, Cairo University, Kasr El-Aini Street, Cairo 11562, Egypt; maha.hegazy@pharma.cu.edu.eg; 3 Department of Clinical Biochemistry, Faculty of Medicine, King Abdulaziz University, P.O. Box 80260, Jeddah 21589, Saudi Arabia; zawan@kau.edu.sa; 4 Department of Pharmaceutics, College of Pharmacy, The Islamic University, Najaf 54001, Iraq

**Keywords:** repurposing, apoptosis, etodolac, zein, HepG2, hepatocellular carcinoma, nanospheres

## Abstract

This work aimed to enhance the purposing profile of Etodolac (ETD) in Human Hepatocellular Carcinoma (HCC) HepG2 cells using sodium deoxycholate stabilized zein nanospheres (ETD-SDZN NSs). ETD-SDZN NSs were formulated using the nan-precipitation method and were characterized, in particular, in terms of mean particle size, zeta potential, encapsulation efficiency, colloidal stability and bioaccessibility. Estimations of cytotoxicity, cellular uptake, cell cycle progression, Annexin-V staining, mRNA expression of apoptotic genes and oxidative stress evaluations were conducted. The ETD-SDZN NSs selected formula obtained an average particle size of 113.6 ± 7.4 nm, a zeta potential value of 32.7 ± 2.3 mV, an encapsulation efficiency of 93.3 ± 5.2%, enhanced bioaccessibility and significantly reduced IC_50_ against HepG2 cells, by approximately 13 times. There was also enhanced cellular uptake, accumulation in G2-M phase and elevated percentage cells in pre-G1 phase, significant elevated mRNA expression of P53, significant reduced expression of Cyclin-dependent kinase 1 (CDK1) and Cyclooxygenase-2 (COX-2) with enhanced oxidative stress by reducing glutathione reductase (GR) level, ameliorated reactive oxygen species (ROS) generation and lipid peroxidation outputs. ETD-SDZN NSs obtained a supreme cell death-inducing profile toward HepG2 cells compared to free ETD. The method of formulation was successful in acquiring the promising profile of ETD in HCC as a therapeutic molecule due to ameliorated cellular uptake, proapoptotic and oxidant potentials.

## 1. Introduction

Despite the huge progress in drug development technologies, there is a highly recommended need to reduce their extended timelines. The utilization of well-known therapeutic molecules having safety records and satisfactory pharmacokinetic profiles will participate in solving this obstacle. The repositioning of clinically safe non-oncological already-approved therapeutic molecules offers additional effective treatment options to cancer patients [1,2]. Another recommendation for drugs repositions the need for influential plans to withstand the generated tumor resistance to used oncological drugs and the suggested related cytotoxicity [3,4].

Hepatocellular carcinoma (HCC) is a prime global health challenge and is estimated to affect more than1 million individuals annually by 2025 [5]. HCC is the main event leading to death in patients with cirrhosis [6]. Additionally, more than 80% of patients who undergo hepatectomy are reported to develop new tumors in the residual liver within 2 years [7]. Dysregulation of the balance between proliferation and cell death constitutes a pro-tumorigenic principle in human hepatocarcinogenesis mainly due to overactivation of anti-apoptotic pathways [8]. Apoptosis inhibition is a vital factor in tumor progression in the pathophysiology of HCC [9]. Distraction of apoptosis pathways may shift cancerous cells to be treatment resistant beside tumorigenesis promotion [10,11]. The induction of apoptosis by anti-carcinogenic molecules has been confirmed with tumor response [11].

Augmentation of apoptosis is a research area that supports cancer treatment procedures. Apoptosis induction in cancerous cells is a main strategy that limits concurrent damage to normal cells [12]. Suppression of apoptosis potential would be a major cause of treatment resistance, unless considering apoptosis the dominant mechanism of antitumor potential [10].

Etodolac (ETD, (R,S) 2-[1,8-diethyl-1,3,4-tetrahydrapyrano(3,4-b)indole-1-yl] acetic acid) is an FDA approved pyranocarboxylic acid-derived COX-2 inhibitor non-steroidal anti-inflammatory (NSAID) drug. ETD is effective in the treatment of ankylosing spondylitis, rheumatoid arthritis, osteoarthritis and in the attenuation of postoperative soreness [13]. Regarding anticancer potential, ETD inhibits peroxisome proliferator-activated receptors (PPAR) and nuclear factor kappa-light-chain-enhancer of activated B cells (NFkB) pathways [14]. ETD has different structural characteristics than other COX-2 inhibitors in that it has no sulfonyl, sulfonamide or sulfone groups to facilitate COX-2 binding [15]. So, it inhibits retinoid X receptor (RXRα) initiating apoptosis in cancer cells with high expression levels of the PPARγ/RXRαnuclear receptor complex. ETD also affects PPAR γfunction which is associated with tumor growth inhibition in mammary glands through the diminishing of cyclin D1. The safety potential of ETD recommends its use in preventative or therapeutic settings due to its post marketing safety data, especially gastrointestinal disturbances being the most frequently listed side effects since its approval in 1999 [16].

Zein (ZN) nanoparticles were recognized as ideal and effective delivery systems throughout therapeutic nanoparticles because of their natural plant origin and adequate method of formulation, which lacks the use of toxic chemical crosslinkers [17]. Zein nanoparticles have exhibited the elevated capability for the extension of plasma levels of different drugs through facilitating its absorption and ameliorating their oral bioavailability [18,19,20,21]. ZN is derived from maize [22], is safe, GRAS (generally recognized as safe), non-toxic, FDA approved in 1985, biocompatible, does not induce celiac disease or autoimmune response and is economic [19,23,24]. ZN nanoparticles exhibited controlled delivery for drug molecules via different administration routes [19,23,24]. ZN is also a protein nanocarrier, so it possesses cytotoxicity, variant renewable sources, and elevated drug-binding capacity [17]. In order to enhance ZN nanoparticles’ colloidal stability, a variety of compounds were suggested to coat ZN nanoparticles such as sodium caseinate [23], sodium deoxycholate monohydrate [25] and dextran sulphate (DS) [26]. DS demonstrated its ability to stabilize ZN nanoparticles in comparison with a variant stabilizer such as polyvinyl alcohol or polysorbates [25].

The aim of this study was to comparatively estimate the anti-proliferation, apoptosis and oxidative stress potentials of ETD-SDZN NSs in comparison to free ETD in HepG2 cells to explore the potential of ZN nanoconstructs in an ETD repurposing profile in HCC management.

## 2. Results and Discussion

**Figure 1:**
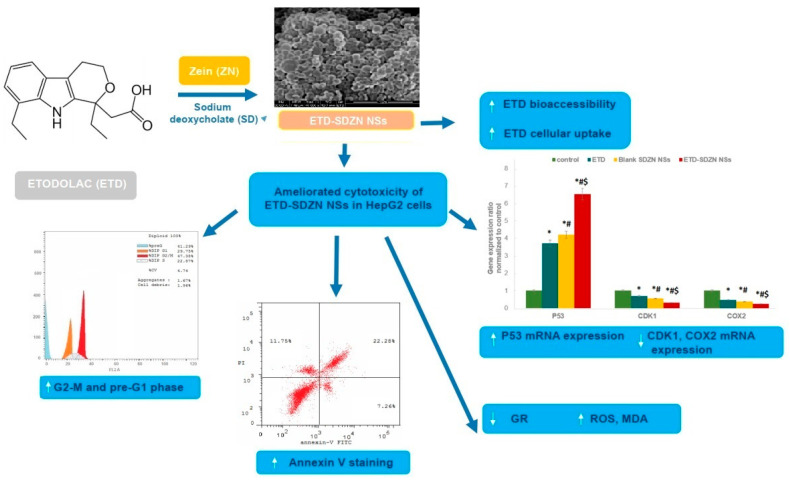
Schematic illustration to summarize all experiments carried out to characterize and evaluate these novel ETD-SDZN Nanospheres. * Significant difference compared to control (*p* < 0.05). ^#^ Significant difference compared to ETD (*p* < 0.05). ^$^ Significant difference compared to SDZN NSs (*p* < 0.05).

The promising anticancer potential of ETD is highly recommended for enhancement. Global tumor resistance has been a great problem leading to apoptosis initiation, and thus treatment failure [27]. The poor water solubility of ETD was also required to be enhanced in order to increase the dissolution rate and the bioavailability [5]. The nanomedicine approach offers distinguished and remarkable amelioration in the delivery of candidate drug molecules in order to support its bioavailability [28], bioaccessibility [29], cytotoxicity [18], apoptosis [30] and repositioning profiles [31]. The present study aimed to develop ZN nano-formulations loaded with ETD in order to obtain fortified potential against HepG2 cells as repositioning molecules. A schematic illustration for all study parts was summarized in Figure 1 in order to present this study in a more specific and reader-friendly way.

### 2.1. Formulation and Characterization of ETD-SDZN NSs

The nano-precipitation method was utilized in the development of ETD nano-formulation according to a planned design including the variable factors. Regarding the molecular structure of ZN, the formation of NSs proceeded as recognizably described by [17], starting with the conformational transition from α-helix to β-sheets, then antiparallel folding of β-sheets to form a toroid ring with a closed center after the encapsulation of therapeutic molecules.

The results of ETD-SDZN NSs characterization were presented in Table 1. As shown from the table, it was noticed that the most diminished mean particle size was associated with formula E7 with a value of 113.6 ± 7.4 nm that was promising to enhance absorption and residence time inside biological systems [23]. Additionally, the obtained reduction in mean particle size could be related to an increase in ZN quantities in the formula. The most elevated particle size magnitude of the E7 formula could be obtained because of the increased quantity of ETD on NSs leading to maximized interfacial tension between NSs’ surface and the aqueous medium; consequently, particle diameter will be increased [20]. In addition, the E8 formula generated the highest magnitude of zeta potential between all the designed formulae (35.6 ± 1.2 mV) while E7 obtained the lowest polydispersity index value (0.19 ± 0.01). The recorded values of zeta potential reflected the potential stability of the formulated ZN nanoplatforms. It was concluded that nanoparticles with a zeta potential above ±30 mV have been recognized as stable structures, as the surface charge postpone agglomeration of developed NSs [32].

The obtained values of encapsulation efficiency (EE) were semi-comparable in the nine formulations. E7 obtained 93.3 ± 5.2% as the highest result. This was in accordance with [18,19] as the binding affinity of drugs on ZN increased with the increase of their polymerization degree. The capability of nano-formulations to incorporate elevated ratios of a loaded drug was previously reported [18,19,20]. This might be as reported [32] in terms of the natural unfolding of ZN molecules at basic pH which offers more reactive sites for enhanced crosslinking and also a noticeable reduction of void spaces within ZN particles. This is expected to positively contribute to enhanced delivery, bioavailability and consequently pharmacological potential. Therefore, the E7 formula was chosen for further investigations.

**Figure 2:**
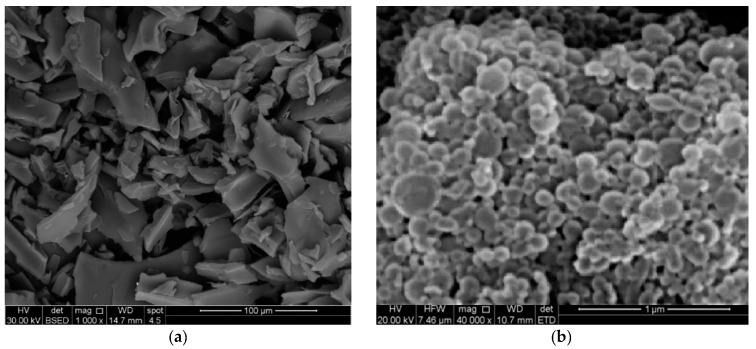
SEM images of (**a**) ZN and (**b**) E7 formula of ETD-SDZN NSs.

The obtained SEM images of the selected formula (E7) displayed discrete nanospheres, which were regular and spherical, discrete, semi-compactly arranged particles with smooth surfaces free of pores or cracks (Figure 2). The measured diameters of the scanned nanospheres were in the range of the obtained mean particle size using a laser diffraction technique (113.6 ± 7.4 nm). The values were 101.9 nm, 99.3 nm and 115.7 nm. They are not the same identically, due to the circumstances of the laser diffraction technique and scanning electron microscopy. In the laser diffraction technique, the nanoparticles were in a liquid state in the cuvette. While in the SEM assay, the nanoparticles were completely dried on a grid. So, there was a possible contraction in the particles measured using SEM, and hence the simple difference in values obtained.

### 2.2. Serum Stability

**Figure 3:**
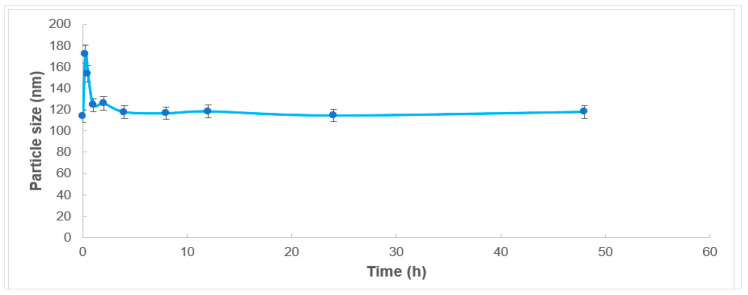
Colloidal stability of ETD-SDZN NSs.

ETD-SDZN NSs’ colloidal stability was evaluated by investigating changes in the mean particle size of the selected formula after incubation in Fetal Bovine Serum (Figure 3). ETD-SDZN NSs’ mean particle size exhibited a preliminary elevation in the first 15 min and then declined quickly to the initial value with non-significant differences. The stabilized size of the prepared nanoplatforms could be due to the potential of SD as a stabilizer. SD was suggested to be adsorbed on ETD-SDZN NSs’ external surfaces through electrostatic or hydrophobic interactions, constituting an interfacial layer on the protein surface of ZN nanoplatforms [25,29]. This approach was suggested to enhance colloidal stability more than electrostatic repulsion [17,33]. The fabricated ETD nanoplatforms displayed a persuaded profile in FBS which proposes a similar pattern in in vivo studies due to the lack of persistent agglomeration generated from imaginable interactions with different physiological molecules inside the biological medium. This conclusion was also in accordance with the suggested use of surfactants such as sodium deoxycholate to develop nanoparticles within the particle size of 100–200 nm with a stabilized colloidal structure [25]. This also could support the suggestion of SD to stabilize ZN nanoplatforms for the delivery of variant therapeuticals.

### 2.3. In Vitro Simulated Digestion Assay

**Figure 4:**
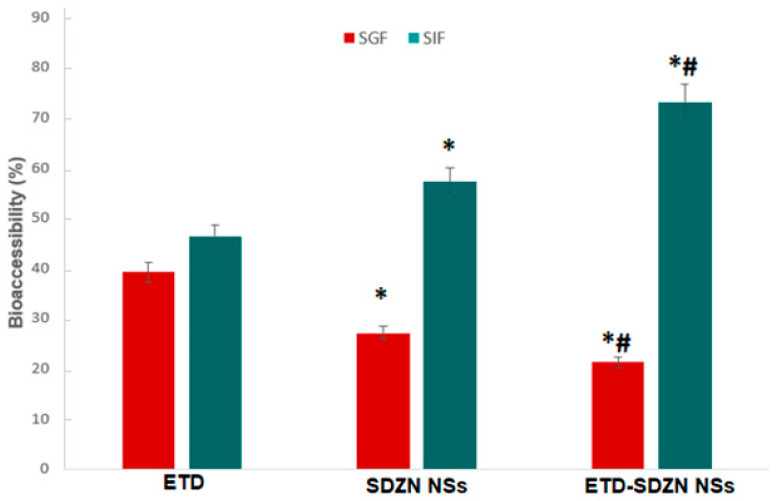
Bioaccessibility of ETD, SDZN NSs and ETD-SDZN NSs in SGF and SIF. Data are expressed as the mean ± SD (*n* = 3). * Significant difference compared to ETD (*p* < 0.05). ^#^ Significant difference compared to SDZN NSs (*p* < 0.05).

In order to overcome the poor water solubility of ETD (about 0.20 mg/mL) and subsequent oral bioavailability and absorption, an in vitro simulated digestion assay via bioaccessibility investigation was proceeded. An estimation of the active drug amount in gastrointestinal tract fluid was analyzed and is presented in Figure 4. ETD bioaccessibility after SIF digestion recorded 46.4 ± 3.7%. ETD-SDZN NSs reflected a significant elevation in the bioaccessibility in SIF (73.1 ± 5.3%) in comparison with ETD and a blank SDZN NSs sample (57.2 ± 3.0%). The concluded results were in harmony with previously reported studies using stabilized ZN NSs in the delivery of anticancer pharmaceuticals [26], which revealed satisfied bioaccessibility due the utilization of ZN delivery systems. The significant elevated bioaccessibility of ETD-SDZN NSs in comparison with SDZN NSs also revealed the influencing patterns of SD as a stabilizing agent for ZN nanoplatforms in the oral delivery of ETD. ETD was able to persist in active form through GIT despite the inclusion of variant components [34] which were considered as the original components of SIF.

### 2.4. Cytotoxicity Assay

**Figure 5:**
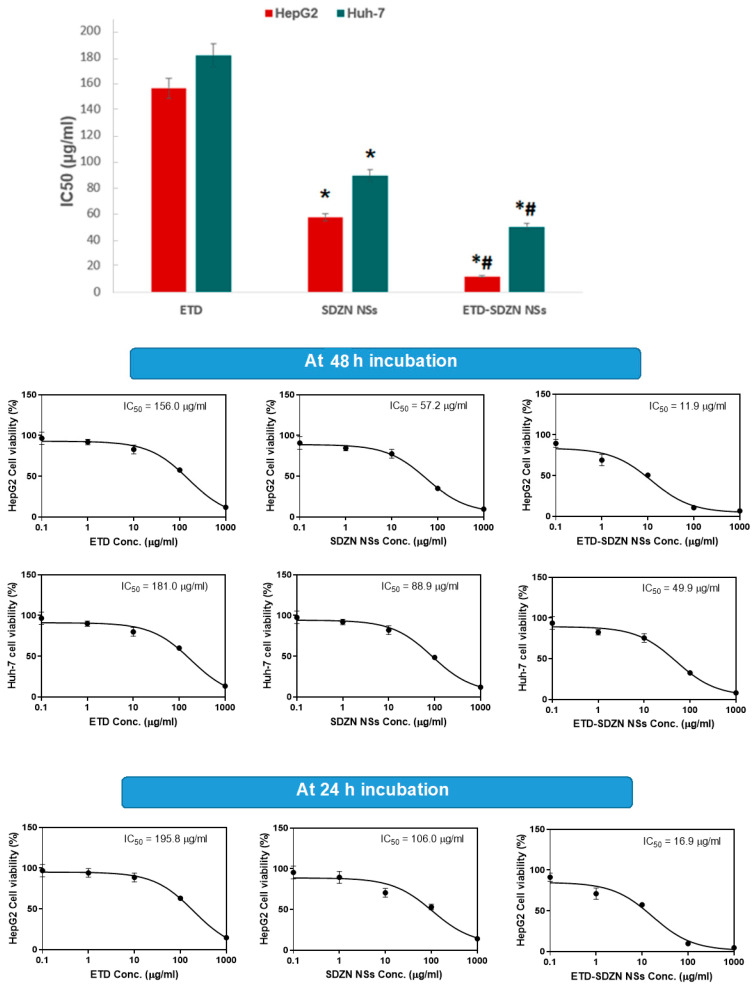
IC_50_ and dose-response curves of ETD, SDZN NSs and ETD-SDZN NSs HepG2 and Huh-7 cells. * Significant difference compared to ETD (*p* < 0.05). ^#^ Significant difference compared to SDZN NSs (*p* < 0.05).

The results of the anti-proliferative profile assay of ETD prepared nanoplatforms are displayed in Figure 5 using mitochondrial function (MTT reduction). The concentrations used in HepG2 cells were 156 ± 11.2 µg/mL, 57.2 ± 3.3 µg/mL and 11.9 ± 0.8 µg/mL for ETD, SDZN NSs and ETD-SDZN NSs, respectively. The concentrations used in Huh-7 cells were 181 ± 14.7 µg/mL, 88.9 ± 5.1 µg/mL and 49.9 ± 2.6 µg/m, respectively, for the same groups. The results reflected superior amelioration of the cytotoxicity pattern of ETD-SDZN NSs in HepG2 and Huh-7 cells in comparison with free ETD. The cytotoxic effect of SDZN NSs in both human liver cancer cell lines due to ZN cytotoxicity was also very remarkable. The minimal value of IC_50_ was generated in HepG2 cells, so we selected HepG2 cells for further investigations. ETD-SDZN NSs obtained enhanced activity of about (IC_50_ = 11.9 µg/mL) compared to free ETD (156.0 µg/mL). This distinguished cytotoxicity confirmed the pattern of SDZN NSs as a promising delivery system due to the remarkably reduced particle size of developed nanoplatforms that can interact effectively on the cellular level with biomolecules. The greater encapsulation within SDZN NSs and the augmented biodistribution of ETD will also support the antiproliferative profile [35,36]. The reported ZN cytotoxicity [18,30] may clarify the acquired cytotoxicity activity of the ETD-SDZN NSs.

### 2.5. Cellular Uptake Analysis

**Figure 6:**
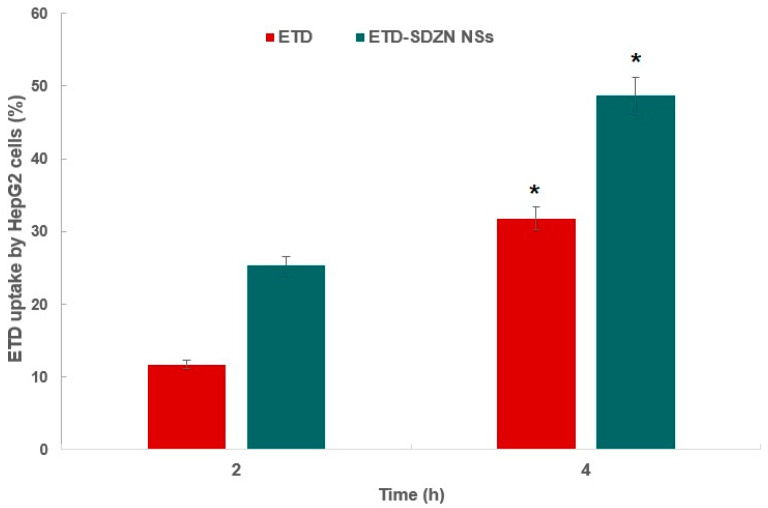
Cellular uptake of ETD by HepG2 cells at 2 and 4 h. * Significant difference compared to uptake at 2 h (*p* < 0.05).

The cellular uptake profiles of ETD by HepG2 cells after exposure to the IC_50_ value of the ETD-SDZN NSs and equivalent weights of free ETD were estimated and are displayed in Figure 6. The cellular uptake of the free ETD was 11.7 ± 0.4% and 31.8 ± 1.6% at 2 and 4 h after starting the incubation, respectively. An elevated uptake was obtained with ETD-SDZN NSs incubations, which reached 25.3 ± 1.9% and 48.7 ± 2.8% after 2 and 4 h of incubation, respectively. The results reflected ameliorated ETD uptake by ETD-SDZN NSs in comparison with free ETD. This also confirmed the SDZN NSs pattern to increase HepG2 cells uptake of ETD, so the pro-apoptotic activity could be ameliorated. It was concluded previously that proline-rich hexapeptide of ZN acts as a cell-penetrating peptide which supports the uptake of pharmaceuticals and different genes by cancerous cells [37]. The suggested mechanism was recognized by an endocytotic mechanism due to the reduced mean particle size of nanostructures (<200 nm) [38]. Additionally, positively charged nanoplatforms yielded an elevated rate of membrane internalization in comparison with negatively charged ones because of enhanced adsorptive-mediated transcytosis triggered by electrostatic interaction between ZN positively charged moieties and cellular membrane negatively charged moieties [39].

### 2.6. Cell Cycle Progression Analysis

**Figure 7:**
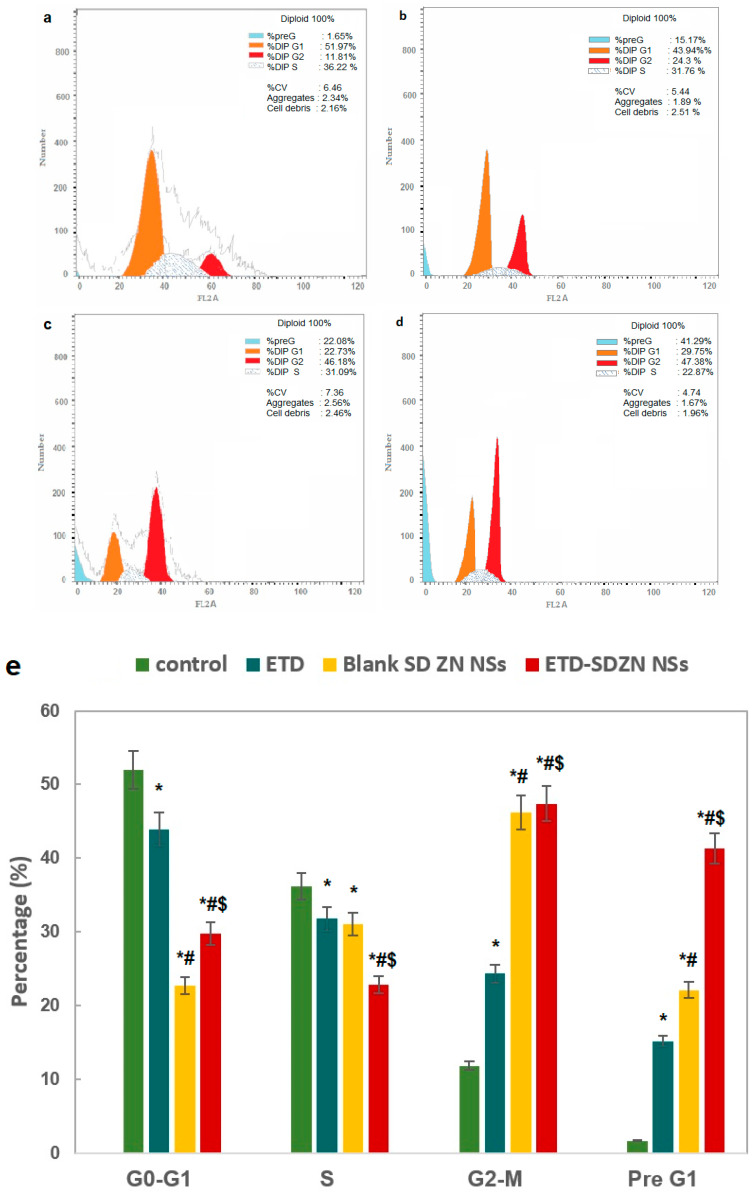
Impact of ETD-SDZN NSs on cell cycle phases. (**a**) Control, (**b**) ETD, (**c**) SDZN NSs, (**d**) ETD-SDZN NSs, (**e**) Graphical presentation of each phase. * Significant difference from control group at *p* < 0.05. ^#^ Significant difference from ETD group at *p* < 0.05. ^$^ Significant difference from SD ZN NSs group at *p* < 0.05.

The control untreated HepG2 cells obtained rapid growth properties, with 51.97 ± 3.9% at the G0/G1 phase, 36.22 ± 1.4% at the S phase, 11.81 ± 0.8% at the G2-M phase, and 1.65 ± 0.2% at the pre-G1 phase (Figure 7a). Other ETD, SDZN NSs and ETD-SDZN NSs incubations exhibited the proliferation of HepG2 cells, especially in the G0/G1 and S phases (Figure 7b–d). Regarding cells’ accumulation in the pre-G phase, 1.65 ± 0.3%, 15.17 ± 0.9%, 22.08 ± 0.9 and 41.29 ± 2.6% were recorded for control HepG2 cells, ETD, SDZN NSs, and ETD-SDZN NSs groups, respectively. The obtained pro-apoptotic pattern may be related to the inhibition of arachidonic acid metabolism through the modulation of COX activity, which influences, to a great extent, proliferation activity in several tumors [40]. The generated ETD pro-apoptosis was also in accordance with the possible roles of COX-2 inhibitors in the inhibition of oral cancer progression [41], breast cancer [40], prostate PC3 cells and colorectal carcinoma HT-29 cells, uterus carcinoma, cervical carcinoma, T cell leukemia [15] and bladder cancer [42]. Figure 7e explains the changes in the different cell cycle phases by graphical illustration.

### 2.7. Annexin-V Assay

**Figure 8:**
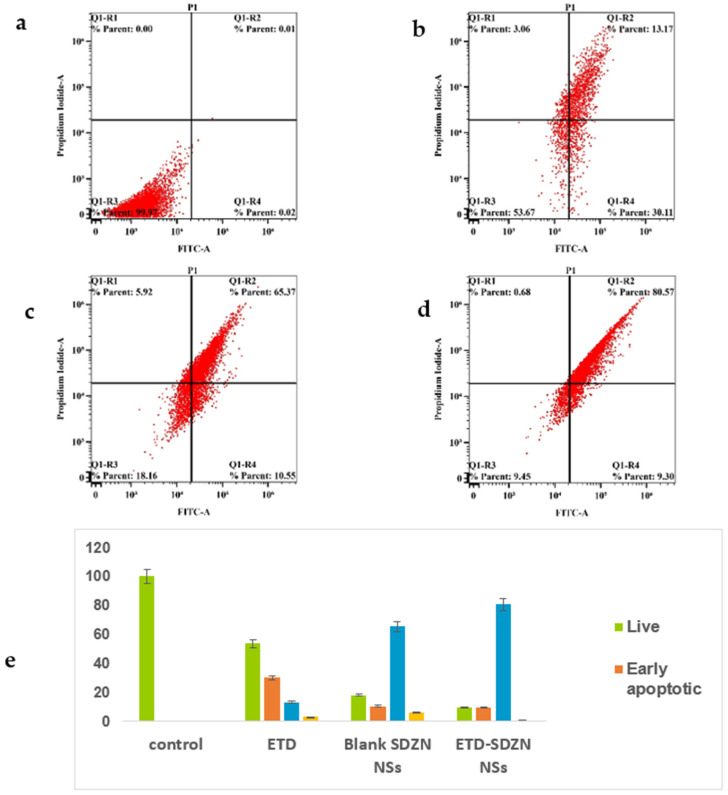
Impact of ETD-SDZN NSs on annexin-V FITC positive staining HepG2 cells. (**a**) Control, (**b**) ETD, (**c**) SDZN NSs, (**d**) ETD-SDZN NSs, (**e**) Graphical presentation of early and late apoptotic, necrotic and total cell death.

After the estimation of cell percentage with positive annexin-V staining in the designed groups’ (control, ETD, SDZN NSs and ETD-SDZN NSs) incubations, results were presented in Figure 8a–d in order to promote the generated apoptotic death of cells. The ETD-SDZN NSs obviously augment the early, late, and total cell death in comparison to all other incubations. Figure 8e showed a graphical estimation of each cell death type.

The developed nanoplatforms—ETD-SDZN NSs—obtained the most potent pattern in augmenting the pre-G phase, which concluded apoptotic cell death confirmed by the annexin-V staining assay. Our results were an argument for late apoptotic death.

### 2.8. mRNA Expression of Apoptosis-Related Genes by Quantitative Real-Time Polymerase Chain Reaction (RT-PCR)

**Figure 9:**
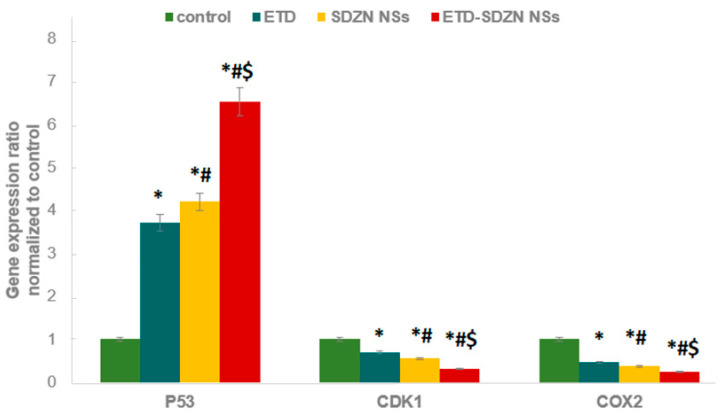
Impact of ETD-SDZN NSs on p53, CDK1 and COX-2 in HepG2 cells. * Significant difference compared to control (*p* < 0.05). ^#^ Significant difference compared to ETD (*p* < 0.05). ^$^ Significant difference compared to SDZN NSs (*p* < 0.05).

The different expression patterns of p53 as a proapoptotic gene and two anti-proapoptotic genes (CDK1 and COX-2) of ETD-SDZNNSs treated cells exhibited statistically different gene expression ratios as displayed in Figure 9. The significant and highest expressions of p53 were obtained in HepG2 cells treated with ETD-SDZN NSs. ETD-SDZN NSs also down-regulated the expression of CDK1 and COX-2 mRNA significantly compared to ETD and SDZN NSs.

p53 protein has been considered a potent transcription factor affecting cell cycle arrest and apoptosis initiation [43], especially in HCC [44]. Different chemotherapeutic agents require p53 to induce apoptosis. Indeed, tumors with a disruption in the p53 pathway are generally resistant to chemotherapy [8]. All groups (ETD, SDZN NSs and ETD-SDZN NSs) displayed changes of 3.71-, 4.2- and 6.53-fold, respectively. ETD-SDZN NSs exhibited higher apoptotic activity in comparison with the control. ETD-SDZN NSs obtained a 1.76-fold elevation in p53 expression in comparison with free ETD. These results were in harmony with that concluded about the ETD profile in different cell lines through a retarding cell cycle or apoptosis generation depending on the p53 profile of cancerous cells [40,45].

Regarding CDK1 expression, there is a relation between CDK1 inhibitors and the generated arrest in G_0_/G_1_ and G_2_/M phases of the cell cycle, especially in tumor cells [46]. Some CDKs inhibitors, in addition to arresting the cell cycle, have a molecular capability to induce apoptosis [47]. The obtained results of CDK1 expression displayed changes of 0.69-, 0.55- and 0.31-fold for ETD, SDZN NSs and ETD-SDZN NSs groups, respectively. ETD-SDZN-NSs displayed an enhanced proapoptotic effect compared with free ETD. ETD-SDZN NSs attained a 2.22-fold reduction in CDK1 level in comparison with free ETD.

COX-2 overexpression is recognized to promote tumor growth via β-catenin stabilization and nuclear translocation leading to growth-promoting genes expression [48]. Thus, the inhibition of COX- is considered to be one of the main mechanisms leading to anticancer potential [49,50]. It will be an integrated value to reduce the side effects of COX-2 expression via apoptosis induction and cell cycle [51]. The obtained results of COX-2 expression showed changes of 0.46-, 0.37- and 0.24-fold for ETD, SDZN NSs and ETD-SDZN NSs groups, respectively. ETD-SDZN-NSs displayed an elevated proapoptotic effect compared with the untreated positive control. ETD-SDZN NSs attained a 1.92-fold reduction in the COX-2 level in comparison with free ETD.

### 2.9. Biochemical Estimation of Oxidative Stress

ZN nanoparticles were considered promising carriers for enhancing the antioxidant potential of several drugs [18,30].

The results of oxidative stress parameters estimation were exhibited in Table 2. ETD-SDZN NSs have the highest capacity to reduce glutathione reduced enzyme (GR) activity, increase reactive oxygen species (ROS) generation and increase Malondialdehyde (MDA) levels in HepG2 cells. Specifically, ETD, SDZN NSs and ETD-SDZN NSs significantly inhibited GR activity by 12.92%, 35.56 and 41.86%, respectively, and compared to the control. This was in accordance with the reported capacity of ETD to enhance the expression of the antioxidant catalase, total glutathione, glutathione peroxidase, glutathione reductase, and superoxide dismutase, which are involved mainly in the initiation and pathogenesis of multiple disease processes [52].

ROS may react with DNA bases to produce oxidative DNA adducts. Such adducts have been associated with mutagenesis and carcinogenesis [53]. As ROS constitute a critical role in apoptosis induction under physiological and pathological conditions, it was reported that ETD may cause increased ROS which in turn activates the apoptotic pathway [54]. ROS generation in HepG2 cells was monitored by DCFH-DA. ETD-SDZN NSs had the highest intensity of green fluorescence. This confirmed that ETD-SDZN NSs could induce the highest oxidative stress in cancer cells by increasing ROS formation via oxidation of NADPH [39]. ETD-SDZN NSs increased ROS formation significantly in comparison with ETD and SDZN NSs. This confirmed the putative scavenging activity against this ROS by anti-inflammatory drugs such as indomethacin, acemetacin, etodolac, tolmetin, ketorolac, oxaprozin and sulindac [52].

MDA summarizes not only its physiological and protective functions as signaling molecule stimulating gene expression and cell survival, but also its cytotoxic role inhibiting gene expression and promoting cell death [55]. ETD, SDZN NSs and ETD-SDZN NSs significantly increase MDA levels by 55.35%, 68.35% and 100.71% as compared to control values, respectively. This attenuation of rise in MDA levels was evidence for the inhibition of lipid peroxidation that extended oxidative stress activity due to the use of SDZN NSs.

## 3. Materials and Methods

### 3.1. Materials and Cell Lines

ETD (CAS number: 41340-25-4), SD (CAS number: 302-95-4), ZN (CAS number: 9010-66-6) and Ethanol were supplied from Sigma-Aldrich (St. Louis, MO, USA). All of the solvents and chemicals used were of analytical grade.

Human hepatocellular carcinoma (HepG2) cells and human hepatocellular carcinoma (HuH-7) cells were supplied by Nawah Scientific, Cairo, Egypt. Cells were cultured in Eagle’s Minimum Essential Medium and Dulbecco’s Modified Eagle’s Medium. All media were supplemented with 10% FBS. Penicillin (100 U/mL) and streptomycin (100 mg/mL) were inserted to flasks and culture plates (SPL Life Sciences, Pocheon, Korea) prior to treatments. Cells were kept at 37 °C in a humidified atmosphere containing 5% CO_2_ (Thermo Electron Corporation, Forma series II, 3141, Beverly, MA, USA) to maintain sub-confluent status of the cells.

### 3.2. Formulation of ETD-SDZN NSs

ZN NSs loaded with ETD were prepared according to the nano-precipitation method [20,29]. Nine ETD-ZN NSs formulae (E1–E9) were prepared using variant ETD:ZN ratios and SD concentration. The correspondent weights of ETD and ZN were dissolved in 10 mL of 85% ethanol with the aid of vortex (Velp scientifica, ZX3, Usmate, Italy) and an ultrasonic probe (Vibra-Cell VCX750; Sonics and Materials Inc., Newtown, CT, USA). The generated ethanolic dispersion was poured into deionized water containing an SD designed concentration and was then 2000 rpm magnetically stirred at room temperature for 3 h to volatilize the ethanol content. The aqueous dispersion was centrifuged at 20,000× *g* speeds and lyophilized without cryoprotectant addition.

### 3.3. Characterization of ETD-SDZN NSs

#### 3.3.1. Particle Size Analysis

The laser diffraction technique was used to measure the mean particle size, zeta potential and polydispersity index of ETD-SDZN NSs formulae. Using a disposable cuvette, one milliliter of the sample was dissolved in deionized water.

#### 3.3.2. Encapsulation Efficiency (E.E.)

Samples were solubilized in ethanol, then filtered through 0.22 µm membrane filters, and subjected to a previously reported high-performance liquid chromatography (HPLC) (Agilent 1200, Agilent Technologies, Santa Clara, CA, USA) equipped with a C18 column, photodiode array detector (PDAD) at 278 nm (Waters, Milford, CT, USA) and adjusted column temperature at 25 ± 2 °C. A mixture of acetonitrile and purified water (50:50 *v*/*v*), pH adjusted to 5.8 with orthophosphoric acid), as a mobile phase with 1 mL/min flow rate according to [56]. The E.E. of ETD was calculated according to the following equation:

(1) $$E.E.\;(w/w\%)\;=\;\frac{\mathrm{amount}\;\mathrm{of}\;\mathrm{ETD}\;\mathrm{in}\;\mathrm{the}\;\mathrm{nano}-\mathrm{constructs}\;}{\mathrm{amount}\;\mathrm{of}\;\mathrm{ETD}\;\mathrm{initially}\;\mathrm{added}}\;\times \;100.$$

#### 3.3.3. Surface Morphology

An SEM instrument (JEM 100-CX; JEOL, Tokyo, Japan) was used to examine the surface morphology of ETD-SDZN NSs. The sample lyophilized powder was fixed initially onto metal stubs and was coated with gold under vacuum.

### 3.4. Colloidal Stability

ETD-SDZN NSs’ colloidal stability was examined in Fetal Bovine Serum (FBS, Gibco, Thermo Fisher Scientific, Waltham, MA, USA) [30]; 1 mL of 70% FBS was added to 200 µL of sample suspension and was then incubated at 37 °C for 48 h followed by magnetic stirring at 600 rpm. At selected time intervals of incubation, particle size analysis was carried out for the sample as measured in Section 3.3.1.

### 3.5. In Vitro Simulated Digestion Assay

The potential of ETD-SDZN NSs and ETD-ZNNSs in enhancing ETD bioaccessibility was investigated using an in vitro simulated digestion assay as reported by [26] using prepared simulated gastric fluid (SGF) and simulated intestinal fluid (SIF). Then, 15 mL free ETD, SDZN NSs or ETD-SDZN NSs were poured into 15 mL of the prepared SGF, and the mixture was adjusted to pH 3.0 using HCl. The prepared mixture was then shaken using a mini-orbital shaker inside the incubator for 2 h at 150 rpm and 37 °C. After that, 15 mL stomach stage mixture was poured into 15 mL SIF, and the dispersion was adjusted to pH 7.0 using NaOH. Then this mixture was shaken for 2 h at 150 rpm at 37 °C. Then, 15 mL of the mixture was cold centrifuged for 40 min at 10,000× *g*. The supernatant was collected and filtered through 0.45 µm membrane filters. ETD content was estimated as mentioned in 3.3.2 ETD bioaccessibility was calculated using the following equation:

(2) $$\mathrm{Bioaccessibility}\;\left(\%\right)=\;\frac{C_{1}}{C_{0}}\;\times \;100.$$

C_1_ is the ETD content in the supernatant and C_0_ is the initial content of ETD in the dispersion.

### 3.6. Cytotoxicity Assay

MTT assay was utilized to evaluate the antiproliferative activity, where HepG2 or Huh-7 cells were seeded into 96-well plates (TPP, Switzerland) approximately as 2 × 10^3^ cells/well. Wells were incubated with ETD, with the equivalent weight of blank SDZN NSs or ETD-SDZN NSs using a range of concentrations with reference to ETD at logarithmic intervals for 48 h at 37 °C in a CO_2_ incubator using 3-[4,5-dimethylthiazole-2-yl]-2,5-diphenyltetrazolium bromide stock solution. A commercially available MTT assay kit was utilized in the estimation of IC_50_ values according to the manufacturer’s instructions (ABCAM, Cambridge, UK).

### 3.7. Cellular Uptake Analysis

In order to determine the magnitude of HepG2 cells uptake, HepG2 cells (1 × 10^5^ cells/dish) were incubated overnight at 37 °C in the presence of 5% CO_2_ for 2 and 4 h after being treated with the IC_50_ value of ETD-SDZN NSs, an equivalent concentration of ETD, as described by [18].

### 3.8. Cell Cycle Progression Analysis

This assay was performed as described by [18,30] using the same cell culture plates, kit, reagents, software and IC_50_ value for 24 h incubation.

### 3.9. Annexin-V Assay

This assay was performed as described by [18,30] using the same cell culture plates, kit, reagents, and IC50 value for 24 h incubation. The apparatus used was Northern Lights 2000 spectral flow cytometer from Cytek Biosciences, USA, together with SpectroFloTM Software version 2.2.0.3.

### 3.10. mRNA Expression of Apoptosis-Related Genes by Quantitative Real-Time Polymerase Chain Reaction (RT-PCR)

This assay was performed as described by [18,30] using the same cell culture plates, kit, reagents, software and IC_50_ value as the used concentration for 24 h incubation. Table 3 presents the primer sequences for P53, CDK1, COX-2 and β-actin. The results were validated using the relative quantification (ΔΔCt) method.

### 3.11. Glutathione Reductase Enzyme Assay

Glutathione reductase enzyme (GR) as a vital oxidative stress marker was evaluated for the developed formulae (ETD, SDZN NSs and ETD-SDZN NSs) using the same reagents and kits described by [18].

### 3.12. Reactive Oxygen Species Assay (ROS) and Malondialdehyde (MDA) Assays

This assay was performed as described by [18,30] using the same cell culture plates, kit, reagents and software.

### 3.13. Statistical Analysis

Study data were presented as the means ± standard deviation (SD) of at least three independent experiments. Comparison between treatments was accomplished by Student’s *t*-test or one-way analysis of variance followed by Tukey’s test (a two-tailed *p* value less than 0.05 was taken as the criterion of significance). Statistical analysis was performed utilizing IBM SPSS software (SPSS Inc., Chicago, IL, USA).

## 4. Conclusions

This present study concluded that ETD has promising repurposing potential as an anticancer molecule against human hepatocellular carcinoma HepG2 cells utilizing pharmaceutical nanotechnology. The functional merger of molecular biology techniques with nanotechnology aspects generated optimistic results. The major results of this study included enhanced cytotoxicity and pro-apoptotic and oxidant profiles related to ETD-SDZN NSs against HepG2 cells in comparison with free ETD and SDZN NSs. In detail, the characterization of ETD nanoplatforms concluded elevated EE% and enhanced bioaccessibility. On the molecular assays, a synergized cytotoxicity potential, augmented uptake by HepG2, supported pro-apoptotic profile, enhanced mRNA expression of P53 and reduced expression of CDK1 and COX-2 genes were obtained comparatively. The RT-PCR results confirmed the apoptosis enhancement obtained by cell cycle assay. Biochemically, the profiles of three oxidative stress markers (GR, ROS and MDA) were evaluated. The obtained results could be as a result of the efficacious utilization of ZN on the biomedical level as a promising biomaterial and effective delivery system for the intensification of an ETD repositioning profile against human hepatocellular carcinoma. The obtained results are recommended for further investigation using in vivo models of hepatocellular carcinoma and clinical studies. This will be very promising regarding the previous safety and approved usage of ETD and ZN by the FDA with beneficial economic costs.

## Figures and Tables

**Table 1:** ETD-SDZN NSs Particle size analysis and encapsulation efficiency.

Formula	ETD:ZN Ratio	SD Concentration (*w*/*v*%)	Mean Particle Size (nm)	Zeta Potential (mV)	Poly-Dispersity Index	Encapsulation Efficiency (%)
E1	3:1	1	265.1 ± 22.1	22.6 ± 1.8	0.67 ± 0.02	81.4 ± 4.9
E2	3:1	2.5	291.3 ±15.7	19.2 ± 1.3	0.51 ± 0.01	87.6 ± 2.8
E3	3:1	5	387 ± 17.8	31.4 ± 1.2	0.38 ± 0.02	88.3 ± 2.3
E4	1:1	1	248.2 ± 15.9	21.1 ± 0.9	0.42 ± 0.02	90.7 ± 4.1
E5	1:1	2.5	277.3 ± 20.8	24.0 ± 1.8	0.36 ± 0.01	92.1 ± 3.6
E6	1:1	5	286.9 ± 23.5	25.9 ± 1.6	0.39 ± 0.02	90.7 ± 5.1
E7	1:3	1	113.6 ± 7.4	32.7 ± 2.3	0.19 ± 0.01	93.3 ± 5.2
E8	1:3	2.5	141.0 ± 5.5	35.6 ± 1.2	0.21 ± 0.01	92.6 ± 5.6
E9	1:3	5	193.4 ± 13.1	31.4 ± 2.7	0.27 ± 0.02	93.0 ± 6.9

**Table 2:** Effect of ETD, SDZN NSs and ETD-SDZN NSs on oxidative stress parameters.

	GR (µU/10^6^ Cells)	ROS (Pg/10^6^ Cells)	MDA (nmol/10^6^ Cells)
Control	1.83 ± 0.16	178.21 ± 11.7	1.39 ± 0.82
ETD	1.62 * ± 0.08	188.49 * ± 9.3	2.16 * ± 0.17
SDZN NSs	1.35 *^,#^ ± 0.38	195.63 * ± 7.8	2.34 *^,#^ ± 0.41
ETD-SDZN NSs	1.29 *^,#,$^ ± 0.09	209.71 *^,#,$^ ± 12.4	2.79 *^,#,$^ ± 0.22

Notes: Data are presented as mean ± SD (*n* = 6). * Significant difference from control group at *p* < 0.05, ^#^ significant difference from ETD group at *p* < 0.05, ^$^ Significant difference compared to SDZN NSs (*p* < 0.05).

**Table 3:** RT-PCR primer sequences.

Primer		Sequence
CASP3	Forward primer	TTCATTATTCAGGCCTGCCGAGG
	Reverse primer	TTCTGACAGGCCATGTCATCCTCA
p53	Forward primer	CCCCTCCTGGCCCCTGTCATCTTC
	Reverse primer	GCAGCGCCTCACAACCTCCGTCAT
CDK1	Forward primer	TGGATCTGAAGAAATACTTGGATTCTA
	Reverse primer	CAATCCCCTGTAGGATTTGG
COX-2	Forward primer	CTCAGACAGCAAAGCCTACC
	Reverse primer	TGACTCCTTTCTCCGCAACA
Β-actin	Forward primer	TCCGTCGCCGGTCCACACCC
	Reverse primer	TCACCAACTGGGACGATATG

## Data Availability

Data is contained within the article.
