# Correction: Kammoun et al. Etodolac Fortified Sodium Deoxycholate Stabilized Zein Nanoplatforms for Augmented Repositioning Profile in Human Hepatocellular Carcinoma: Assessment of Bioaccessibility, Anti-Proliferation, Pro-Apoptosis and Oxidant Potentials in HepG2 Cells. *Pharmaceuticals* 2022, *15*, 916

**DOI:** 10.3390/ph18040505

**Published:** 2025-03-31

**Authors:** Ahmed K. Kammoun, Maha A. Hegazy, Alaa Khedr, Zuhier Ahmed Awan, Maan T. Khayat, Majid Mohammad Al-Sawahli

**Affiliations:** 1 Department of Pharmaceutical Chemistry, Faculty of Pharmacy, King Abdulaziz University, P.O. Box 80260, Jeddah 21589, Saudi Arabia; 2 Analytical Chemistry Department, Faculty of Pharmacy, Cairo University, Kasr El-Aini Street, Cairo 11562, Egypt; 3 Department of Clinical Biochemistry, Faculty of Medicine, King Abdulaziz University, P.O. Box 80260, Jeddah 21589, Saudi Arabia; 4 Department of Pharmaceutics, College of Pharmacy, The Islamic University, Najaf 54001, Iraq

## Text Correction (1)

There was an error in the original publication [1]. There was an error in 3.9. Annexin-V Assay, the flow cytometer was not identified. A correction has been made to 3. Materials and Methods, 3.9. Annexin-V Assay.

This assay was performed as described by [18,30] using the same cell culture plates, kit, reagents, and IC50 value for 24 h incubation. The apparatus used was Northern Lights 2000 spectral flow cytometer from Cytek Biosciences, USA, together with SpectroFloTM Software version 2.2.0.3.

## Text Correction (2)

There was an error in the second paragraph of 2.7. Annexin-V Assay, our results were an argument for early and late apoptotic death and total cell death, which influence the pattern of ETD to induce apoptosis in HCC cells. A correction has been made to 2. Results and Discussion, 2.7. Annexin-V Assay.

The developed nanoplatforms—ETD-SDZN NSs—obtained the most potent pattern in augmenting the pre-G phase, which concluded apoptotic cell death confirmed by the annexin-V staining assay. Our results were an argument for late apoptotic death.

## Error in Figure 8

In the original publication, there was a mistake in Figure 8 as published. The corrected Figure 8 appears below. This mistake may have originated from mislabeling of the annexin-V figures and subfigures in my digital archive.

The authors state that the scientific conclusions are unaffected. This correction was approved by the Academic Editor. The original publication has also been updated.

## Figures and Tables

**Figure 8:**
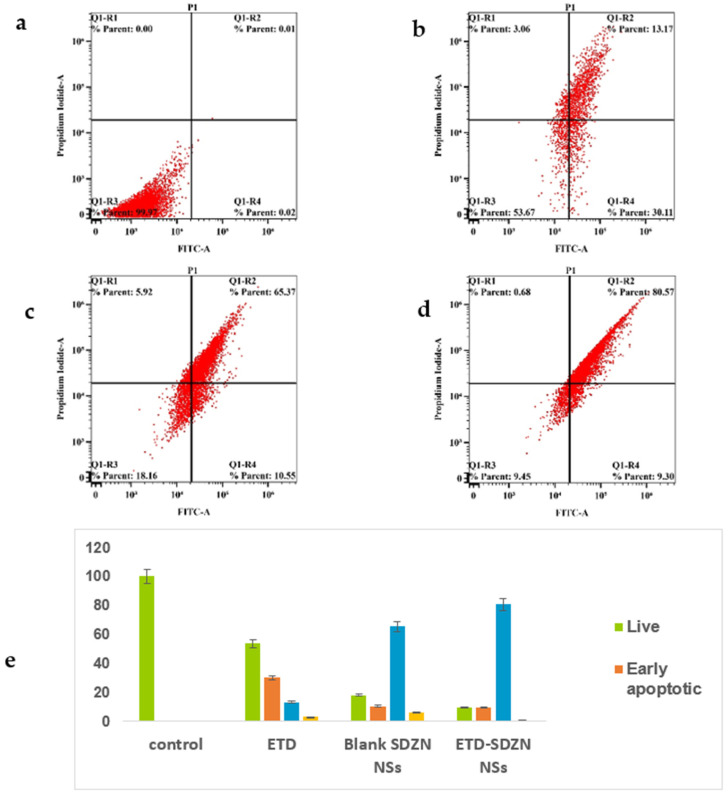
Impact of ETD-SDZN NSs on annexin-V FITC positive staining HepG2 cells. (**a**) Control, (**b**) ETD, (**c**) SDZN NSs, (**d**) ETD-SDZN NSs, (**e**) Graphical presentation of early and late apoptotic, necrotic and total cell death.
